# Editorial: Progress in the application of biomaterials and nanotechnology in cell biology

**DOI:** 10.3389/fcell.2025.1679902

**Published:** 2025-08-19

**Authors:** Liqiang Zhou

**Affiliations:** ^1^ Faculty of Health Sciences, University of Macau, Macau, Macao SAR, China; ^2^ MoE Frontiers Science Center for Precision Oncology, University of Macau, Macau, Macao SAR, China

**Keywords:** nanotechology, biomaterials, regenerative medicine, drug delievery, nanomaterials (A)

The integration of biomaterials and nanotechnology is revolutionizing cell biology, as exemplified by the pioneering studies in this Research Topic. Exosome-mediated co-delivery of curcumin and methylene blue (Yang et al.) demonstrates synergistic neuroprotection in Alzheimer’s models, leveraging exosomes’ blood-brain barrier permeability to modulate autophagy and reduce amyloid-β toxicity. Simultaneously, mechanobiology insights emerge from adipose regeneration research (Ye et al.), where dynamic force transduction activates YAP/β-catenin signaling, guiding scaffold design for transplanted adipocyte survival. Complementing therapeutic innovation, fluorescent carbon dots (CDs) within PEC-GS/BG hybrids (Zhang et al.) achieve real-time cellular tracking with minimal cytotoxicity, exemplifying how nanoscale optical probes illuminate intracellular dynamics. Theranostic convergence is further evident in miRNA-nanomedicine systems (Telkoparan-Akillilar et al.), where tumor-targeted nanoparticles exploit endogenous regulatory pathways for combinatorial gene/chemotherapy, while precision nanomedicine advances (Mao et al.) highlight biomarker-responsive nanomaterials enabling patient-specific cancer diagnosis and treatment.

Collectively, these studies underscore certain transformative trends, including multifunctionality in nanocarriers, bidirectional material-cell communication, and closed-loop diagnostic-therapeutic integration. Future progress hinges on decoding nano-bio interfaces at single-cell resolution and establishing scalable biomaterial manufacturing (Liu et al., 2023). As spatial omics and AI-driven design mature, next-generation platforms will likely transcend traditional compartmentalization, enabling real-time adaptation to cellular microenvironments (Jiang et al., 2025). This Research Topic crystallizes a pivotal shift toward predictive and participatory nanomedicine-where materials actively collaborate with biological systems to restore function.
